# Lung microbiome: new insights into bronchiectasis’ outcome

**DOI:** 10.3389/fcimb.2024.1405399

**Published:** 2024-06-04

**Authors:** Alice Azoicai, Ancuta Lupu, Monica Mihaela Alexoae, Iuliana Magdalena Starcea, Adriana Mocanu, Vasile Valeriu Lupu, Elena Cristina Mitrofan, Alin Horatiu Nedelcu, Razvan Tudor Tepordei, Dragos Munteanu, Costica Mitrofan, Delia Lidia Salaru, Ileana Ioniuc

**Affiliations:** ^1^ Mother and Child Medicine Department, “Grigore T. Popa” University of Medicine and Pharmacy, Iasi, Romania; ^2^ CF Clinical Hospital, Iasi, Romania; ^3^ Faculty of Medicine, “Grigore T. Popa” University of Medicine and Pharmacy, Iasi, Romania

**Keywords:** lung, bronchiectasis, microbiome, bacteriome, virome

## Abstract

The present treatments for bronchiectasis, which is defined by pathological dilatation of the airways, are confined to symptom relief and minimizing exacerbations. The condition is becoming more common worldwide. Since the disease’s pathophysiology is not entirely well understood, developing novel treatments is critically important. The interplay of chronic infection, inflammation, and compromised mucociliary clearance, which results in structural alterations and the emergence of new infection, is most likely responsible for the progression of bronchiectasis. Other than treating bronchiectasis caused by cystic fibrosis, there are no approved treatments. Understanding the involvement of the microbiome in this disease is crucial, the microbiome is defined as the collective genetic material of all bacteria in an environment. In clinical practice, bacteria in the lungs have been studied using cultures; however, in recent years, researchers use next-generation sequencing methods, such as 16S rRNA sequencing. Although the microbiome in bronchiectasis has not been entirely investigated, what is known about it suggests that *Haemophilus*, *Pseudomonas* and *Streptococcus* dominate the lung bacterial ecosystems, they present significant intraindividual stability and interindividual heterogeneity. *Pseudomonas* and *Haemophilus*-dominated microbiomes have been linked to more severe diseases and frequent exacerbations, however additional research is required to fully comprehend the role of microbiome in the evolution of bronchiectasis. This review discusses recent findings on the lung microbiota and its association with bronchiectasis.

## 1 Introduction

The majority of human pathogenic processes, including infections, cancer, autoimmune diseases, and atopies, are significantly influenced by the microbiome, which is thought to be more complicated than the human DNA. Therefore, it has become a heavily researched topic in the last ten years due to its importance in understanding the physiopathology of many diseases and in the development of future therapeutics. Some microbiome components may be particularly harmful to human health, and new research has linked viruses and bacteria to long-term inflammation, which is particularly dangerous in lung disease (Amon, 2017). Respiratory illnesses are one of the most common pathologies in the world, with a significant financial burden on the health care system and are a major cause of morbidities and mortality Consequences and clinical manifestations of the chronic lung disease bronchiectasis are diverse. The gradual progression of the disease may be attributed to the interplay among chronic infection, inflammation, and impaired mucociliary clearance, all of which contribute to structural alterations and the emergence of new infections. The interaction among these components facilitates the progression of the disease (Keir and Chalmers, 2021). The microbiome associated with bronchiectasis remains largely unexplored; however, the available data indicates that lung bacterial communities consist primarily of *Pseudomonas*, *Haemophilus*, and *Streptococcus*. These communities exhibit significant variability between individuals and remain stable within them (Richardson et al., 2019). In this narrative review we present the recent developments concerning the role of the lung microbiome in bronchiectasis.

Bronchiectasis, a heterogeneous clinical and etiological disease, from genetic or acquired conditions is characterized by permanent airway dilatation and wall thickening (Li et al., 2024). The outcomes are still unknown, the treatment focuses on treating the aggravation. The optimal duration of antibiotic treatment for bronchiectasis exacerbations has not been systematically studied, 14 days’ therapy being extrapolated from the treatment cystic fibrosis (Scioscia et al., 2019).Therefore, it is critical to study the clinical aspects, pathogenesis and treatment of bronchiectasis, as well as the pulmonary microbiome and its various properties, to better understand the correlation between these two entities.

## 2 Human microbiota/respiratory tract microbiota

Despite the significant and ongoing interest in the gut microbiome, there has been a growing body of evidence in the last years suggesting that local interactions with lung bacteria characterize the pathophysiology and immunology of the respiratory tract (Ashbaugh et al., 1967; Ranieri et al., 2012).

Pathogenic, commensal, and symbiotic microorganisms (microbes, viruses, protozoa, and fungus) coexist in our bodies to form organ-specific microbial communities, which make up the human microbiota. The microbiome varies in size and structure depending on the body area and is influenced by both the host and external variables. While some species of the microbiome are beneficial to human health, others are regarded to be particularly hazardous: specific bacteria and viruses have been connected to chronic inflammation, which raises the risk of lung disease (Dominguez-Bello et al., 2019).

The entire genome of all the microorganisms that reside in or on a specific host or tissue is referred to as the microbiome. Because of their diversity, the inability of certain bacteria to be cultivated on different media, and the need for genetically based research, every ecosystem has a distinct population of microorganisms. It is well recognized that assessing and estimating microorganisms in their natural habitats can be challenging at times (Aleman and Valenzano, 2019). Although the colonization of the respiratory system, particularly the lungs, by bacteria, fungi, and viruses may not always result in a cytopathological effect, the existence of non-pathogenic microbes has significantly improved the body’s overall health (Barko et al., 2018). Lung colonization exhibits greater diversity, although lower microbial load compared to other tissues and organs in the body. The lung microbiome is difficult to study and characterize by conventional laboratory techniques, however, lifestyle, underlying disease, and the abuse of antibiotics have been some of the problems in assessing the lung microbiome (Belizário and Faintuch, 2012).

**Figure 1:**
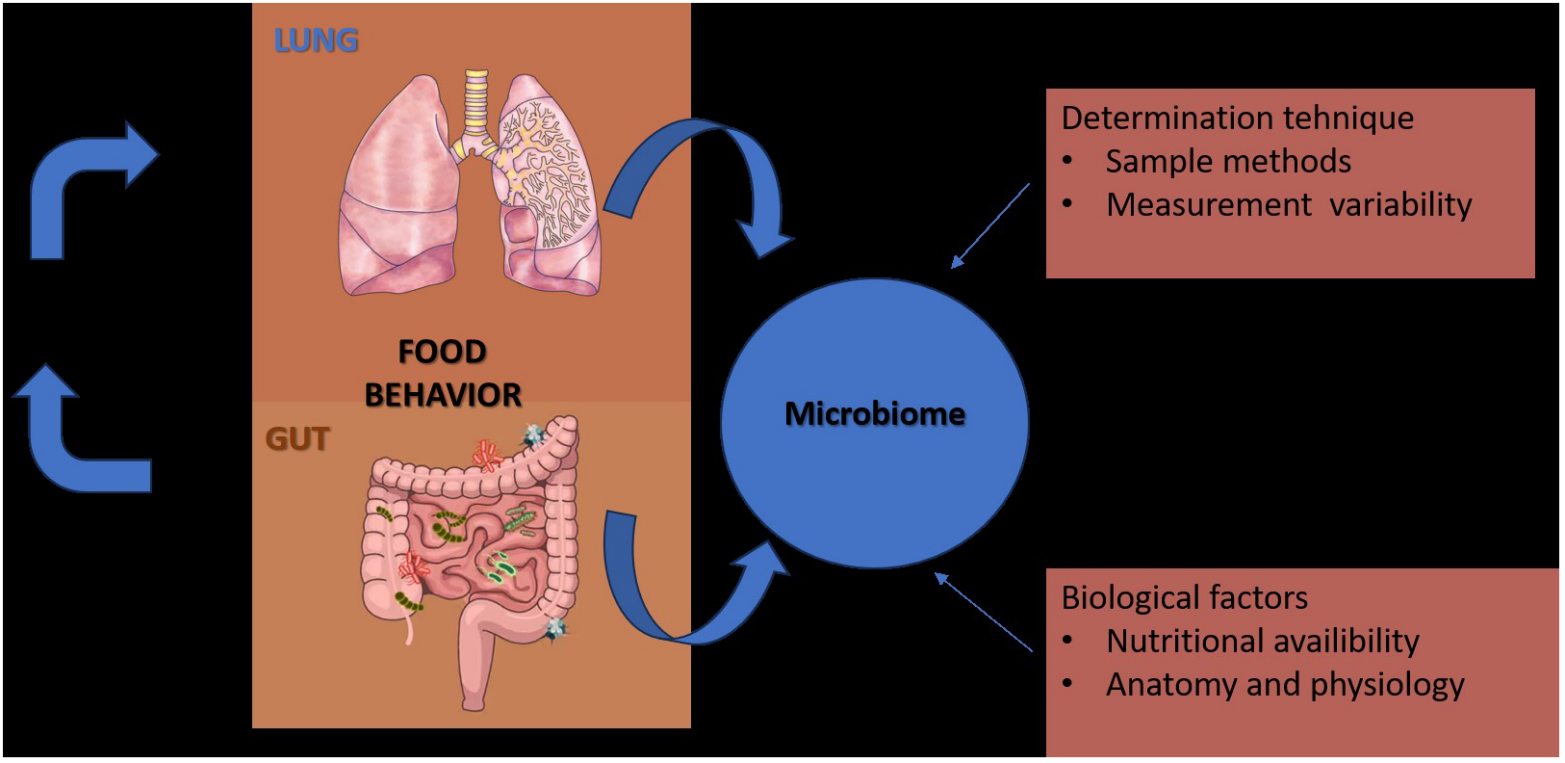
Factors that contribute to the diversity of results in studies of the gut and lung microbiome.

In contrast to the gut, which is merely a long lumen with relatively simple and unidirectional bacterial transit (Lagier et al., 2012), the lungs are depicted as a dividing tree with airways expanding to the 70 m^2^ surface area of alveoli and bacterial movement that is tidal and bidirectional (by cough, a constant immigration by inhalation and microaspiration, and a constant efflux via mucociliary clearance) (Calfee et al., 2014; Sinha et al., 2018). Because of the structure of the respiratory system, practical considerations (invasiveness of the procedure, requirement for anesthesia), procedural worries (such the possibility of contamination and the microbial biomass of the specimen), there is no “gold standard” specimen for studying the respiratory microbiome. Till the last century was promulgated the theory that “the normal lung is free from bacteria” (Cotran et al., 1999), but following the dawn of germ theory (1898–1929), the published studies of lung microbiology, reported that viable bacteria could be cultured from the lungs of humans and large animals, that thousands of viable bacteria are inhaled each hour, and that subclinical microaspiration of upper airway secretions is common among healthy persons underlying the fact that the lungs and upper respiratory tract are under constant exposure to the microorganisms of inhaled air (O’Dwyer et al., 2019) (**Figure 1**).

There are three conceptual fallacies that stand for the lung sterility, important for the practitioners to know (Dickson et al., 2014b; Dickson et al., 2014a):

1. Pathogenic bacteria during infections were intended to be detected by laboratory microbiologic culturing procedures, not the typical respiratory microbiota.
2. The incorrect concepts of ecological proximity and contamination.
3. There is a misconception that “absence of resident microbiota” and sterility are synonymous. In fact, the balance between immigration and elimination is the only factor that distinguishes microbial and non-microbial populations.

While every respiratory disease has a unique combination of trigger events and pathogenic mechanisms, all respiratory diseases share fundamental traits, such as epithelial injury or malfunction, airway inflammation, and airway remodeling. The majority of treatments only temporarily relieve symptoms; they do not, therefore, effectively prevent sickness, which decreases people’s quality of life (Richardson et al., 2019).

**Figure 2:**
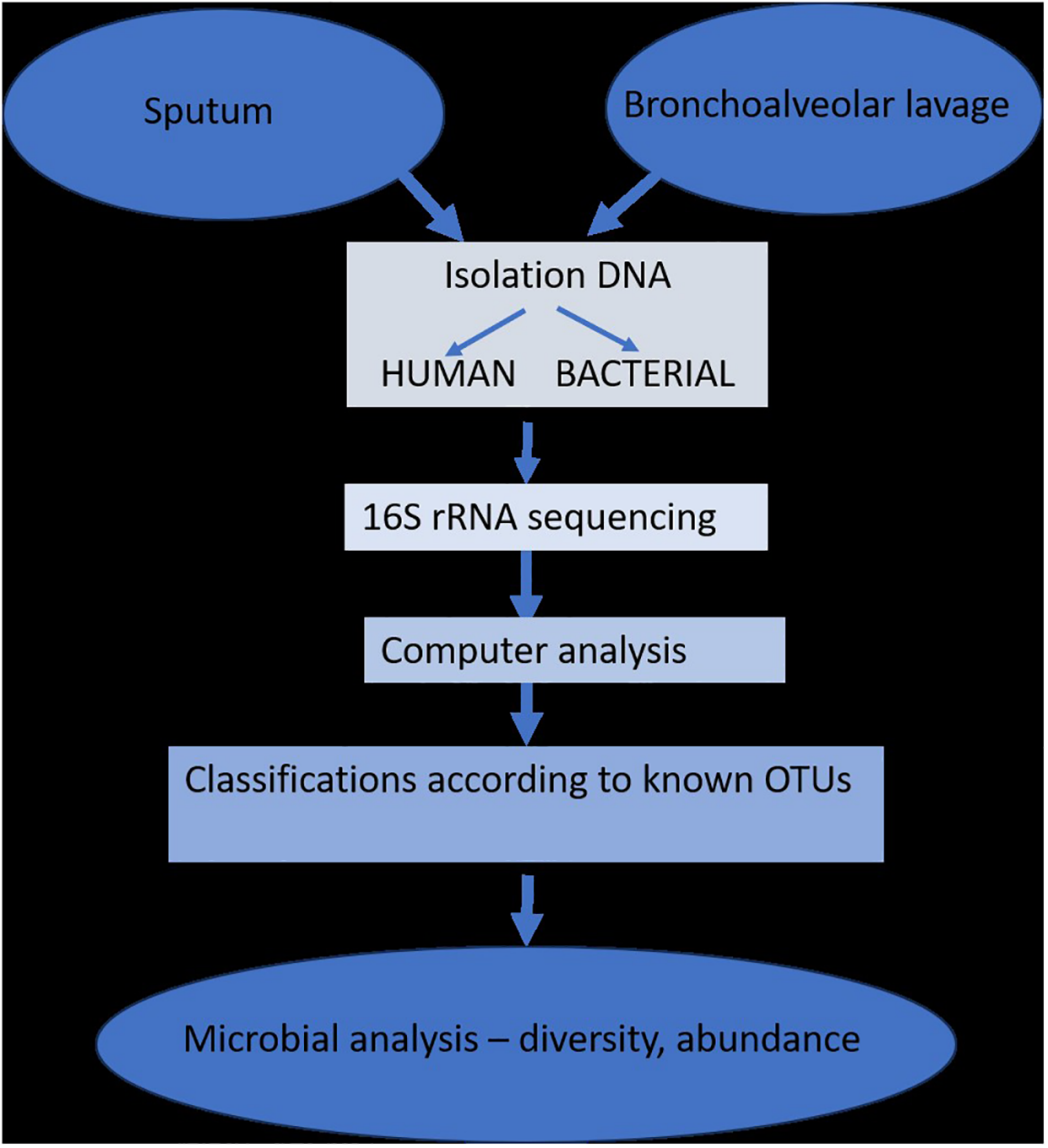
The isolation and determination of lung microbiota (adapted from Dickson et al., 2014b).

Since most studies have demonstrated that changes in the respiratory microbiome may have an impact on the development of respiratory disorders, the biology of the lung microbiome has the capacity to distinguish between commensals, pathogenic, non-pathogenic, and opportunistic bacteria. The study of lung microbiota has been successfully accomplished through DNA hybridization techniques, 16S rRNA sequencing, metagenomics, and other specialized techniques performed on sputum or bronchioalveolar lavage fluids. However, the research is heavily influenced by changes brought about by age, diet, lifestyle, or antibiotic use (**Figure 2**) (Dickson et al., 2014b).

**Figure 3:**
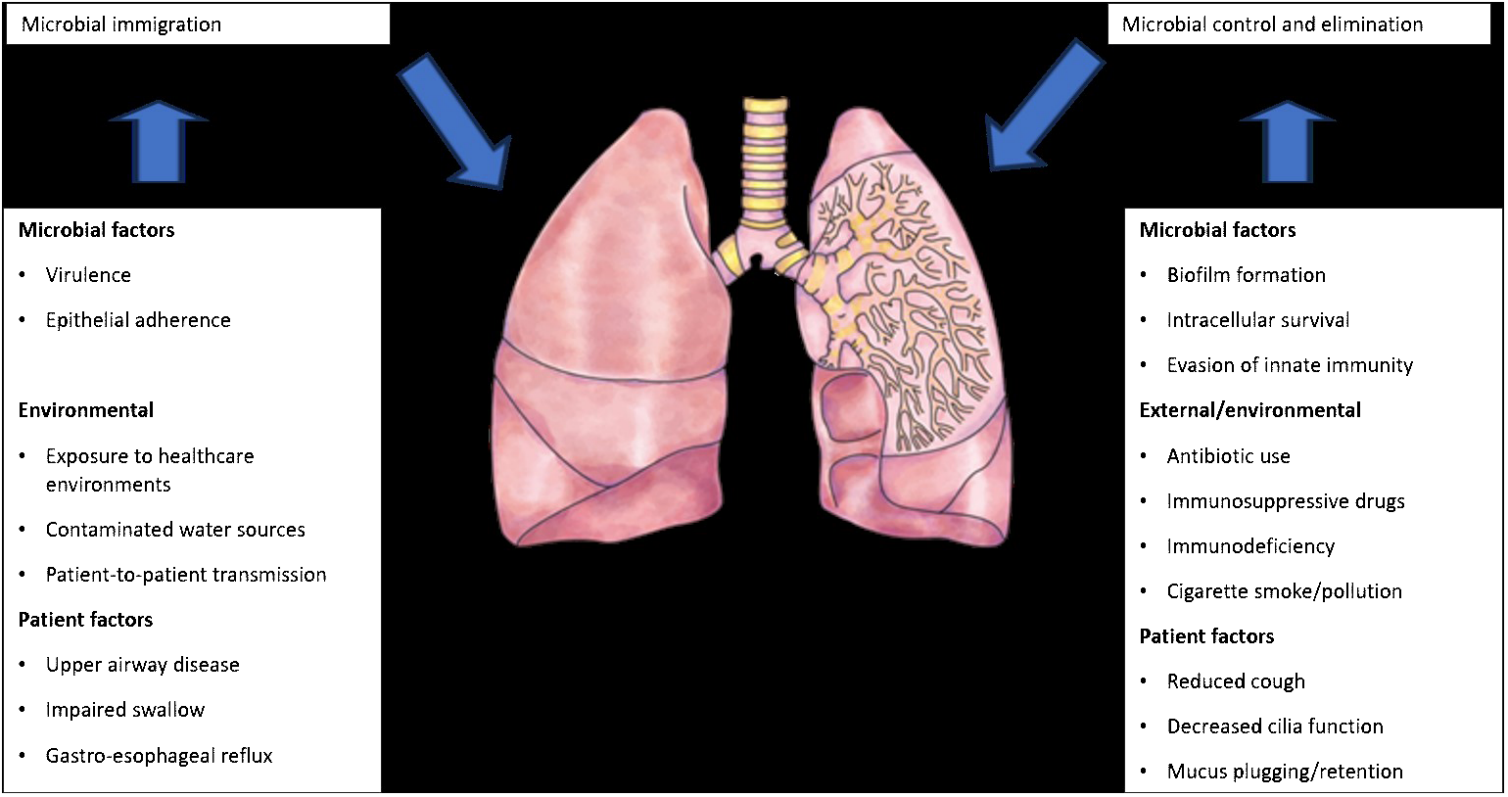
Factors that can modify the lung microbiome (adapted from Richardson et al., 2019).

The introduction of next-generation sequencing technologies has sparked renewed interest in respiratory tract microbiome investigations in recent years, despite the difficulties and considerable obstacles that still persist in exploring the lung microbiome despite the availability of instruments. There are two main sources of false signal in respiratory microbiome researches: sampling contamination and sequencing contamination (e.g. bacterial DNA present in laboratory reagents): while bronchoalveolar lavage (low biomass specimen) is less susceptible to sampling contamination but more susceptible to reagent contamination, sputum (high biomass specimen) is more susceptible to sampling contamination due to pharyngeal microbiota and less susceptible to sequencing contamination. However, under strict control, bronchoscopic studies have shown that, provided suitable measures are taken (e.g., reducing suction through the main channel before lavage), pharyngeal bacteria have little effect on bronchoalveolar lavage and protected specimen brushing specimens (Bilen, 2020) (**Figure 3**). Despite the fact that contamination from both sequencing and sampling presents considerable methodological challenges, study design is universal and does not depend on the kind of material (Davenport et al., 2017): reduce systematic bias in specimen processing and sampling; incorporate a large number of negative controls (sequencing and procedural); openly disclose all sequencing data, including negative control data; validate significant findings by complementary assays, replicated experiments, and contextual plausibility (Richardson et al., 2019). Most common approaches for sampling the respiratory microbiome are: bronchoalveolar lavage, tracheal aspirate, protected specimen brushing, sputum, surgically resected and explanted lung tissue, upper respiratory tract swabs.

### 2.1 Use of antibiotics

It has been demonstrated that overuse of antibiotics alters the lung microbiota and causes pulmonary diseases to develop. Proteobacteria are the most common microbes in a healthy rat lung, closely followed by a smaller proportion of certain phyla like *Firmicutes*, *Bacteroidetes*, and *Actinobacteria*. The impact of using the antibiotic Levofloxacin on the lung microbiota of laboratory rats was examined in a study by Finn et al. (2019), which highlights the antibiotic’s potent activity against the majority of commensal bacteria in a healthy subject. While the lungs of animals that were not treated, showed a mixed bacterial flora, primarily belonging to the genus *Serratia*, the lungs of animals that received treatment primarily, contained bacteria from the genus *Pantoea*. This study hypothesized that irrational usage of antibiotics affects the ecology of the microbiota via a reduction in bacteria diversity and the reports of Barfod et al. (2015) that Vancomycin possesses the ability to preferentially disrupt murine lung microbiota underlines this theory. It is underlined that inhaled *Pseudomonas. agglomerans* endotoxins could determine the activation of alveolar macrophages and secretion of mediators such as interleukin-1, tumor necrosis factor (TNF), and prostaglandins, that lead to the accumulation of platelets in pulmonary capillaries triggering acute and chronic inflammation and later to bronchoconstriction, reduced forced expiratory volume in the first second, reduced diffusing capacity of the lung for carbon monoxide, and increased airway reactivity, which leads to significant respiratory symptoms (Shrestha B et al., 2021).

### 2.2 Tobacco smoke

The components of the human microbiome are susceptible to a number of stimuli, such as food, alcohol, smoking, and antibiotics, despite the fact that it is stable and capable of recovering after deregulation. In addition to directly interacting with the bacteria in the lung, smoking affects the immune system, promotes the growth of biofilms, and alters oxygen tension in the lung microbiome (Huang and Shi, 2019). Free radicals, transition metals, contaminants, reactive oxygen species, and many other tobacco constituents all contribute to the negative effects by creating a complex cocktail of carcinogenic and toxic potentials (Larsson et al., 2008).

Numerous microbes have been found in new tobacco leaves, including *Stenotrophomonas maltophilia*, *Pseudomonadaceae species* including *Pseudomonas fluorescens*, and *Pantoea agglomerans* as well as *Acinetobacter calcoaceticus* (Mehta et al., 2008). Since Sapkota et al. (2010) found a variety of bacteria in cigarettes, including human commensals and soil-dwelling *Pseudomonas aeruginosa, Acinetobacter, Clostridium, Klebsiella, and Burkholderi*a, we can conclude that smokers may acquire and colonize bacteria in various ways due to their lifestyle choices. Beyond this, given that tobacco smoke has a devastating effect on the peripheral immune response, resulting in decreased natural killer cell activity and an increased susceptibility to infection, it is possible that the different bacterial load and population in addicted smokers is caused by decreased host cell defenses as a result of tobacco’s immunosuppressive nature.

In addition, smoking is linked to a decrease in airway dendritic cells and an increase in macrophages, neutrophils, eosinophils, and mast cells, which alters macrophage and neutrophil activity (Droemann et al., 2005). Additionally, some bacteria may be encouraged to form biofilms by cigarette smoke (Kulkarni et al., 2012). Studies on inflammation caused by smoking should take lung microbiota variation into account, as Zhang et al. (2018) found in their study that smoking impacts both the microbial population and diversity of the LRT. Smoking is involved in the raising incidence of lung diseases (Lupu et al., 2023).

## 3 Microbiome and bronchiectasis

### 3.1 Bronchiectasis

Chronic, irreversible bronchial dilatation along with thickening of the airway walls due to a breakdown of bronchial elastin and supporting tissue structures is the hallmark of bronchiectasis. Recurrent respiratory infections and a persistent cough with persistent sputum production are the major clinical signs. Increased inflammation brought on by these infections is linked to lung function reduction, dyspnea, and damage to the airways (Chalmers et al., 2018). Clinically, malaise, pain in the chest, hemoptysis, and weight loss are possible additional symptoms (Mac Aogáin and Chotirmall, 2019). High-resolution computed tomography (HRCT) is the gold standard for diagnosing it; note the morphological subtypes as well (Mac Aogáin and Chotirmall, 2019): cylindrical, common and characterized by smooth tubular bronchi and mild disease; varicose, non-uniform dilation; cystic, associated with more severe disease and complete loss of bronchial morphology.

Patients experience airflow limitation despite their airways appearing to expand visibly. This is because to poor bronchial secretion drainage and blockage in the small and medium airways, which is mostly caused by inflammatory and viral assaults. The microbial colonization of the lung is ensured by impaired mucociliary clearance and mucus inspissation, in addition to other detrimental effects (Flume et al., 2018). The principal etiology is indicated by: post-infectious bronchiectasis, immunodeficiency diseases, obstructive lung disease, ciliary disorders, genetic disorders, skeletal diseases, obliterans bronchiolitis, idiopathic (Chalmers et al., 2018; Chandrasekaran et al., 2018; Flume et al., 2018; Mac Aogáin and Chotirmall, 2019; Lupu et al., 2023).

**Figure 4:**
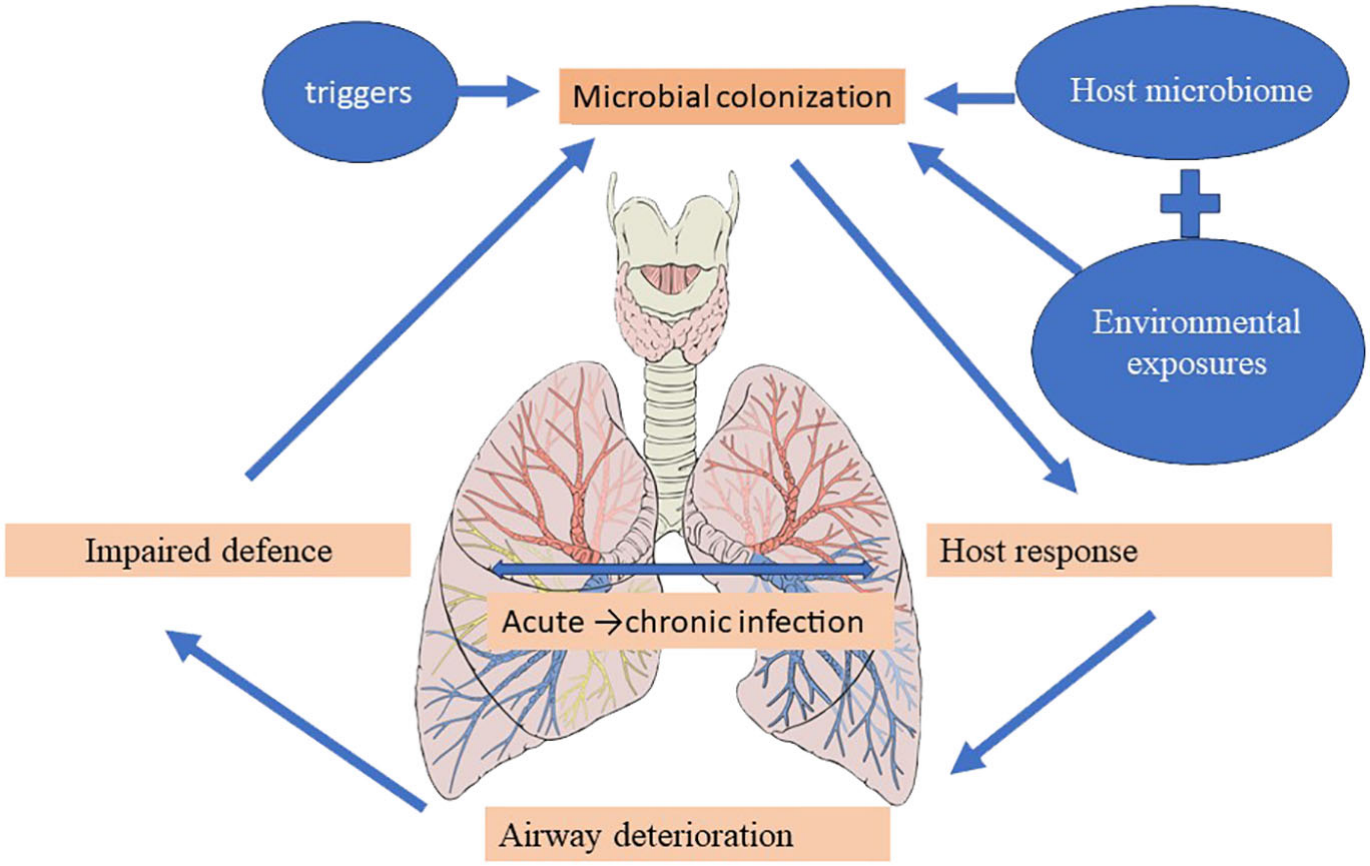
Bronchiectasis’ Pathogenesis.

The vicious cycle hypothesis, initially presented by Cole, postulates that trigger factors linked to genetic susceptibility and host defense deficiencies determine a self-reinforcing cycle of inflammation, infection, and impaired mucociliary clearance, culminating in a progressive dilatation and destruction of the bronchial wall (Cole, 1986). Airway insults from recurrent childhood infections predispose to the development of bronchiectasis in the future (Wurzel et al., 2016), and the high incidence of bronchiectasis at the extremes of age is accompanied by significant shifts in the underlying immune status and the lung microbiome (Chotirmall and Burke, 2015; Tamburini et al., 2016). The presentation of pediatric bronchiectasis differs from that of adult cases. Following Cole’s original theory, more intricate and comprehensive models—like the recently developed “vicious vortex” paradigm put forth by Flume et al. (2018)—emphasize the dynamic interactions dictated by each pathophysiologic stage of this disease cycle, given the long-term, persistent inflammation and progressive damage to the airways. Accordingly, the microbiome may be a key player in the etiology and course of disease, offering predictive potential for better patient outcomes in a clinical environment (**Figure 4**) (Rogers et al., 2014b; Chalmers and Chotirmall, 2018; Richardson et al., 2019).

Understanding the pathogenesis of bronchiectasis and possible therapeutic strategies for its clinical management can be based on a culture-based assessment of the local microbial pathogens and the features of the immune responses linked with it (King et al., 2007).

### 3.2 Infection’s role

The most common pathogens found in culture-based studies to be associated with bronchiectasis are *Pseudomonas aeruginosa, Haemophilus influenzae, Streptococcus pneumoniae, Moraxella catarrhalis, and Staphylococcus aureus* (King et al., 2007; Foweraker and Wat, 2011; Chalmers and Chotirmall, 2018; Chandrasekaran et al., 2018). *Mycobacterium tuberculosis* is also a significant post-infective etiology (Chandrasekaran et al., 2018; Dhar et al., 2019). A higher chance of *Aspergillus fumigatus* fungal colonization and an exacerbation of pre-existing bronchiectasis are linked to non-tuberculous mycobacteria (NTM) infection (Kunst et al., 2006; Aksamit et al., 2017; Chotirmall and Martin-Gomez, 2018). A poor prognostic sign is the occurrence of fungal infections such as *Candida, Penicillium, Cryptococcus, Clavispora, and Scedosporium*, particularly when allergic bronchopulmonary aspergillosis (ABPA) develops (Chandrasekaran et al., 2018; Chotirmall and Martin-Gomez, 2018; Mac Aogáin et al., 2018; Mac Aogáin et al., 2019a; Cuthbertson et al., 2021).. Although the exact involvement of viruses in bronchiectasis is unknown, viruses such as influenza, rhinovirus, and coronavirus have been often found in individuals with bronchiectasis (Gao et al., 2015; Wurzel et al., 2016; Chalmers and Chotirmall, 2018; Mitchell et al., 2018).

Last but not least, the so-called contaminated oropharyngeal flora might cause an unfavorable immunological reaction by directly inciting inflammation or by influencing other microbial structures (Segal et al., 2016; Layeghifard et al., 2019). We could think of the microbiome-associated bronchiectasis as a dynamic structure that affects lung structures through the generation of elastases, cytokines, and matrix metalloproteinases (MMPs) in an unregulated manner. Immune responses triggered by the activation of microbial structures are often neutrophilic and less frequently eosinophilic. Elevated levels of IL-1β, IL-8, leukotriene (LT)B4, CXCL2, and TNFα are consistently present in heterogeneous cytokine responses (Angrill et al., 2001; Fuschillo et al., 2008; Taylor et al., 2015; Chalmers et al., 2017). These levels support the release of intercellular adhesion molecule-1 (ICAM-1) and vascular cell adhesion molecule-1 (VCAM-1) from the endothelium, which in turn promotes the recruitment of neutrophils and eosinophils into the airways (Fuschillo et al., 2008; Chalmers and Hill, 2013). The function of the neutrophil remained the most important.

Despite a longer survival and delayed apoptosis, the neutrophil produces proteases as neutrophil elastase, which represent a negative prognostic indication and are functionally hampered, impairing bacterial phagocytosis and killing (Bedi et al., 2018; Chotirmall, 2018). A thorough mechanistic analysis of neutrophils serves as the foundation for the development of Brensocatib, an inhibitor of neutrophilic serine protease activation, which is the subject of a phase II clinical trial in the future (Chalmers and Chotirmall, 2018; Chalmers et al., 2020). Neutrophils’ dysregulated function in bronchiectasis is also linked to changes in the microbiome’s makeup, with *Proteobacteria—including Moraxella, Pseudomonas, Enterobacteriaceae*, and *Stenotrophomonas*—predominating (Finch et al., 2019).

### 3.3 The airway microbiology in bronchiectasis

#### 3.3.1 Bacteriome

Over time, certain microbiological organisms indicative of bronchiectasis have been described, mostly based on cultures, with varying prevalence according to geographic location. The most often found bacterial species are *Haemophilus influenzae and Pseudomonas aeruginosa*, followed by *Streptococcus pneumoniae, Moraxella catarrhalis, Staphylococcus aureus, and Klebsiella pneumoniae*. Less frequently discovered are *Stenotrophomonas maltophilia* and *Achromobacter xylosoxidans* (Foweraker and Wat, 2011; Finch et al., 2015). Even in the presence of significant inflammatory responses, no microbial pathogen has been found in approximately 70% of bronchiectasis sputum cultures (Fuschillo et al., 2008; Richardson et al., 2019). For this reason, the microbiome’s genetic analyses linked to next-generation sequencing (NGS) are very helpful in understanding the microbiologic pattern in bronchiectasis. The investigation of the lung microbiome without regard to culture begins with cystic fibrosis (CF). A larger cohort from a clinical trial of macrolide intervention was recently subjected to 16S rRNA sequencing by Rogers et al. in the BLESS research, underscoring this as a potentially informative analytical marker in respiratory trials (Rogers et al., 2014a). In comparison to the control group, the BLESS intervention (low-dose erythromycin, 400 mg twice day) reduces exacerbations, and 16S rRNA analysis sheds light on the composition of the microbiome as a correlate of the observed therapeutic response. Macrolides improve clinical outcomes in inflammatory lung disorders, especially in patients with non-CF bronchiectasis who experiment biofilm pathology (Serisier et al., 2013). Nevertheless, erythromycin was found to be more effective in eliminating bacterial pathogens, and macrolides have demonstrated non-traditional antimicrobial effects, such as changing the morphology of *P. aeruginosa*-in particular, and preventing adherence to the respiratory epithelium when exposed to erythromycin *in vitro* (Tsang et al., 2003). Erythromycin also dramatically decreased the formation of sputum in this category of patients. As shown in *in vitro* investigations, this may indicate a direct reduction of mucus secretion (Shimizu et al., 2003); however, decreased mucus formation is likely also an expected byproduct of antibacterial or anti-inflammatory actions (Kohri et al., 2002).

By including microbiome analysis, patient categorization based on the dominant organism was made possible, revealing fine-grained variations in microbiome composition. Patients having dominating profiles of *Veillonella* or *Pseudomonas* had a markedly inferior result (Rogers et al., 2014b; Rogers et al., 2014a). *Veillonella*’s link to exacerbation may indicate that this anaerobe plays a deceitful function in the intricate microbial community associated with bronchiectasis (Rogers et al., 2014b). Moreover, there is a correlation between the microbiome profiles and the host immune response. Specifically, *Haemophillus influenzae* has been shown to enhance MMP2 and MMP8 in individuals with pseudomonas-dominant profiles, while both bacteria have been shown to trigger significantly higher levels of CRP in serum and IL-1β and IL-8 in sputum. The BLESS cohort, in general, emphasizes the stability of the bronchiectasis microbiome and notes the substitution of more pathogenic *Pseudomonas. aeruginosa* strains for *Haemophillus. influenzae*. Depending on the makeup of the microbiome, even the effect of erythromycin therapy can be mostly positive. Although the benefits of this medication were associated with a higher risk of future *Pseudomona*s colonization in non-*Pseudomonas*-dominant patients, it did not significantly change exacerbation rates in Pseudomonas-dominant patients. Similar to individuals with severe asthma treated with macrolides, the whole-genome metagenomic shotgun sequencing analysis describes a gradual development in macrolide resistance in patients receiving antibiotics. A core macrolide resistome is still present in respiratory specimens from healthy individuals, according to further study (Taylor et al., 2018; Taylor et al., 2019; Mac Aogáin et al., 2020). It is unclear how macrolides, which have no action against P. aeruginosa, provide this therapeutic benefit in bronchiectasis; potential explanations include the drug’s anti-inflammatory and immunomodulatory properties, as well as indirect effects on other microbial components (Huffnagle et al., 2017). Microbiomes have the potential to predict clinical outcomes and therapeutic responses; nevertheless, stability during exacerbation and therapy is a crucial aspect of microbiome research in bronchiectasis (Rogers et al., 2014a; Cox et al., 2017; Richardson et al., 2019), particularly in light of potential future therapeutic benefits. Contrary to the oversimplified theories that propose the targeted antibiotic removal of pathogenic microorganisms as the foundation for treatment success, individual microbiomes tend to remain relatively stable over time. It’s possible that some antimicrobial drugs, like macrolides, which have no effect on *P. aeruginosa*, indirectly affect other microbial components and the community structure they’re associated with, or they may have immunomodulatory and anti-inflammatory effects (Zemanick et al., 2017).

#### 3.3.2 Virome

The most difficult and recently discovered component of the human microbiome is the virome (King et al., 2007); the function of viruses in bronchiectasis is still unknown, despite the fact that they have been found in the airway and may play a part in the disease due to their effects on health status, their influence on bacterial hosts, and the resulting acquired immunodeficiency (Gao et al., 2015; Mitchell et al., 2018; Chen et al., 2020b). In a cohort of patients with bronchiectasis, Gao et al. evaluated the presence of coronavirus, rhinovirus, and influenza A and B viruses at exacerbation. While the symptoms of virus-positive and virus-negative people were similar, there were differences in various systemic and airway inflammatory indicators (serum IL-6 and TNF-α; sputum IL-1β and TNF-α) among the former group (Gao et al., 2015). The rhinovirus and influenza A and B had the largest effects, and Chen et al. subsequently discovered a significantly increased frequency of viruses at exacerbation compared to the steady condition (Chen et al., 2020b). A further possible function of viruses could be represented by acquired immune insufficiency, such as that caused by the Epstein-Barr virus or the Human T-cell leukemia virus type 1, which can shorten normal immunological homeostasis and speed up the disease “cycle” (Chen et al., 2020a; Normando et al., 2020). The role of bacteriophages and their contribution to the architecture and stability of the microbiome have been the subject of recent studies due to their importance in the context of emerging antimicrobial resistance and their ability to facilitate horizontal gene transfer, which is crucial in respiratory diseases like bronchiectasis (Rolain et al., 2011).

#### 3.3.3 Mycobiom

Fungal sensitization and allergy are linked to detrimental effects in bronchiectasis, similar to other chronic respiratory diseases: lower lung function and higher exacerbation (Nguyen et al., 2015; Chotirmall and Chalmers, 2018). For patients with cystic fibrosis, Aspergillus fumigatus is a significant airway fungal infection linked to a loss in lung function. Fungal infections are still difficult to diagnose because of their low sensitivity and specificity, which can cause therapy delays and unfavorable results (Delhaes et al., 2019; Mac Aogáin et al., 2019b). A decreased fungal diversity has been reported in bronchiectasis associated with cystic fibrosis in comparison to non-CF bronchiectasis (Cuthbertson et al., 2021). When compared to healthy controls, the Cohort of Asian and Matched European Bronchiectasis (CAMEB), the primary study of the bronchiectasis mycobiome, revealed a higher abundance of potentially pathogenic fungi such as *Aspergillus, Penicillium*, and *Cryptococcus.* Additionally, the study revealed the presence of an undesirable allergic sensitization associated with Aspergillus. Geographical regions were then used to identify distinct mycobiome profiles: Asians had larger abundances of *Wickerhamomyces, Clavispora*, and *Cryptococcus*, whereas Europeans had higher abundances of *Simplicillium, Trichosporon*, and *Aspergillus*. In both generations, Candida was often observed (Mac Aogáin et al., 2018; Tiew et al., 2020).

#### 3.3.4 Multibiome

The bacteriome, mycobiome, and virome—which were previously thought of as distinct entities—are now combining to form a comprehensive “multibiome” that affects bronchiectasis as well as other lung disorders. Future research on the microbial interactions related with respiratory illness infection and exacerbation, particularly those involving fungus and bacteria as well as the impact of viruses associated with acquired immunodeficiency, may provide valuable insights (Layeghifard et al., 2017).

#### 3.3.5 Other microbiome

While studying the lung microbiome in chronic respiratory disorders is typical, it’s also crucial to consider the makeup of the microbiomes in other anatomical sites. The makeup of the oral microbiome may impact the lower airway’s immune system and highlight the existence of other respiratory disorders (Huffnagle et al., 2017; Yuan et al., 2020). The possible micro-aspiration of gut bacteria and the ensuing inflammatory effects could alter the composition of the gastrointestinal microbiome. The pathogenic response to microbial contacts in the lung may be influenced by the connection via the lung-gut axis between immunological homeostasis and the gut microbiota (Bacher et al., 2019; Budden et al., 2019). As comorbidities with bronchiectasis, gastroesophageal reflux disease and irritable bowel syndrome suggested the potential for a dysbiotic gut microbiota (Deshpande et al., 2018).

The limited number of patients (often less than 150 patients) and short follow-up periods (less than a year) have limited the studies on the microbiome in bronchiectasis (**Table 1**).

**Table 1:** Microbiome research in bronchiectasis.

No	Study	Method	Biome	Clinical correlation
1.	Rogers et al. (2014a)	Targeted 16SrRNA analysis	Bacteriome	Exacerbation frequency
2.	Taylor et al. (2018)	Targeted host gene sequencing 16S rRNA analysis	Bacteriome	Exacerbation frequency, lung function
3.	Taylor et al. (2019)	WGS metagenomics	Bacteriome	Antimicrobial resistance
4.	Mac Aogáin et al (Mac Aogáin et al., 2018; Mac Aogáin et al., 2019a)	Targeted ITS analysis	Mycobiome	Exacerbation frequency, lung function
5.	Tiew et al. (2020)	WGS metagenomics	Mycobiome	Exacerbation frequency
6.	Gao et al. (2015)	Polymerase chain reaction assays	Virome	Exacerbation frequency
7.	Chen et al. (2020b)	Targeted qPCR respiratory virus panel	Virome	Odds ratio for exacerbation/time to next exacerbation

## 4 Future perspectives

The fact that most bronchiectasis therapy trials have been unsuccessful can be partly attributed to the increasing understanding of the diversity of clinical endophenotypes in this condition (Chalmers and Chotirmall, 2018). In addition to concentrating on microbial endophenotypes, the microbiome offers potential as an additional outcome measure and prognostic indicator when assessing the effects of treatment targeted at it. Monitoring any potentially detrimental alterations in the microbial community, like the emergence of potentially pathogenic taxa or the existence of genes associated with antibiotic resistance, is also advantageous (Taylor et al., 2018; Mac Aogáin et al., 2020).

Host genetics is another crucial factor that, when accessible, at least partially predicts the microbiome’s makeup and ought to be taken into account when developing patient classification algorithms (Taylor et al., 2017). New insights have already been discovered by expanding the examined microbial kingdoms in bronchiectasis through the use of multi-biome analysis. One such finding is the identification of fungal sensitization, which could be connected to precision medicine methods that target endophenotypes (Mac Aogáin et al., 2019b). Furthermore, the early application of metagenomics to bronchiectasis treatment has demonstrated significant potential for exposome mapping and the development of antibiotic resistance. While the precise role of the bronchiectasis virome remains unclear, much research conducted recently suggests a potential association with the likelihood of exacerbations.

The discovery of unique, persistent phage profiles in gut microbiomes has provided a clear framework for future lung studies, especially those looking at bacteriophages in bronchiectasis. Still, a broad evaluation of the virome, or “phageome,” linked to the bronchiectasis microbiota is necessary.

The air microbiome and the environment’s microbiome are increasingly understood to be important components of respiratory health and are most likely connected to bronchiectasis (Tiew et al., 2020). Bronchiectasis and other chronic respiratory disorders are known to be exacerbated by air pollution and can result in hospital admissions (Garcia-Olivé et al., 2018; Landrigan et al., 2018). Additionally, there is a substantial correlation between sensitization and bronchiectasis, indicating the possibility of host-environment interactions and the potential use of metagenomic study (Mac Aogáin et al., 2019b).

While assessing the lung microbiome in chronic respiratory illnesses is a reasonable place to start, consideration should also be given to the microbiome composition of other anatomical regions. Because it can influence or predict the lower airway’s immune status or possibly the presence of further respiratory illnesses, the oral microbiome is significant. Together with the upper and lower respiratory tracts, the oral microbiome creates an ecological gradient (Huffnagle et al., 2017; Yuan et al., 2020).

The composition of the gut microbiome is especially important because of the potential for sub-clinical micro-aspiration of gut bacteria and the ensuing inflammatory repercussions. Additionally, the relationship between immunological homeostasis and the gut microbiota via the lung-gut axis may also have an impact on the lung’s pathogenic reaction to microbial interactions (Bacher et al., 2019; Budden et al., 2019). Gastrointestinal disorders including inflammatory bowel disease and gastroesophageal reflux disease, which have both been found to be comorbidities with bronchiectasis, may be signs of a dysbiotic gut microbiota (Lloyd-Price et al., 2019).

Further research is necessary to ascertain whether the lung microbiome and clinical presentation are connected in order to aid choose the optimal course of treatment, even though studies on the lung microbiome have not yet affected clinical practice. Future microbiome study has to include a larger number of patients and a longer follow-up time in order to completely capture the heterogeneity of the illness both during stabile state and during exacerbation.

It is unlikely that microbiome sequencing would significantly alter clinical practice in its current form because it is a very time-consuming process that need for specialized bioinformatic analysis. Third-generation sequencing techniques such as Oxford Nanopore Technologies’ MinION and PacBio’s single-molecule real-time sequencing may be used in clinical practice. The UK’s INHALE research project used the MinION in the past to quickly diagnose pneumonia acquired in hospitals and related with ventilators, as well as to analyze lower respiratory tract infections microbiologically (Richardson et al., 2019).

## 5 Adult versus pediatric lung microbiome

Regardless of the age, infection plays a key role in the etiology of bronchiectasis and is based on theories such as the “vicious cycle” and “vicious vortex” (Mac Aogáin et al., 2021). Globally, *Pseudomonas aeruginosa* and *Haemophillus influenzae* are the most commonly found bacteria in bronchiectasis airways, both in children and adult patients, although their relative abundance varies among populations of microorganisms. In comparison to *Haemophillus influenzae*, *Pseudomonas aeruginosa* is linked to increased hospitalizations, worse lung function, and higher rates of morbidity and mortality. This confirms that the lung microbiota and the degree of the disease have a close correlation, mostly in pediatric population. Several investigations have demonstrated that an increase in exacerbations causes an increase in airway and systemic inflammation, which is linked to a rapid deterioration in lung function (Preda et al., 2022).

In both adult and pediatric patients, antibiotics directed against bacteria reduce bacteria burden, the resulting inflammation, and the chance of worsening, which reduces symptoms and improves treatment outcomes (Mac Aogáin et al., 2021). Still, the fact that in certain cases we are unable to identify microbial species that are generally not regarded as clearly hazardous by more conventional criteria serves as an example of how complex and lacking our understanding of the distinction between pathogen and pathobiont in bronchiectasis is (Mac Aogáin and Chotirmall, 2022). In the context of bronchiectasis, for instance, a certain organism may be considered beneficial or even normal in a healthy airway, but it may take on a more detrimental role (Mac Aogáin and Chotirmall, 2022). This is a justified reason for continuing research in the field of lung microbiota in patients with bronchiectasis.

## 6 Microbiome and chronic disease in pediatric pathology

The subject of lung biology is starting to reveal new insights into the microbial landscape of the respiratory tract through innovative techniques and technology. It is now evident that the human lungs are often exposed to live bacteria and their byproducts, despite the fact that the lungs were once thought to be a sterile environment. Interestingly, the lung microbiome has a low biomass and is driven by dynamic fluxes of microbial immigration and clearance. It is becoming more and more clear as we gain knowledge of the microbial ecology of the lung that certain disease states can disrupt the microbial-host interface and ultimately affect disease pathogenesis (Natalini et al., 2023).

Majority of the lung microbiome studies that have been published to date use an observational research strategy that compares cross-sectional data with clinical factors. As mentioned in other reviews, studies on the lung microbiome have confirmed that the microbial composition of respiratory diseases, including asthma, chronic obstructive pulmonary disease`, bronchiectasis, lung cancer, and respiratory viral infections, differs from that of healthy subjects. However, descriptions of the lung microbiome alone do not fully explain the underlying mechanisms of these diseases (Cox et al., 2019; Carney et al., 2020). As a result, each study design should be evaluated in light of reliable and accepted experimental methods, feasibility, and sample principles.

### 6.1 Lung microbiome and asthma

The lung microbiome itself may not be useful as a treatment in respiratory disorders, even though the gut microbiome may be changed by diet to change the gut-lung axis. For example, while randomized, controlled trials have not demonstrated a decrease in the incidence of asthma, the use of probiotics that restore a healthy gut microbiome may potentially lessen Th2 cytokine responses and improve symptomatology in pediatric patients (Wei et al., 2020; Yagi et al., 2021).

There is mounting evidence that the development of asthma is significantly influenced by the microbiome of the airways. Early infancy appears to be a crucial time for the development of a highly diversified, non-pathogenic bacterial community, but it is also a “window of opportunity” for modifying the upper airway microbiota and the immune system in order to potentially shield children from developing asthma. The pathophysiology and severity of adult asthma are influenced by dysbiosis of the airway microbiota (Hufnagl et al., 2020; Yagi et al., 2021). Numerous environmental factors can have an impact on the microbial composition of the gut and lung. Asthma can be exacerbated by environmental stressors such pollution, viruses, allergies, and the use of PPIs or antibiotics that lead to bacterial dysbiosis. However, in a farm setting, exposure to other bacterial components as well as proteins could be protective against the development of asthma. Preventive and therapeutic management to counteract microbiome dysbiosis and restore a healthy microbiome by probiotics, fecal microbiota transplants or bacterial lysates has not arrived in clinical routine so far. Thus, further mechanistic studies are needed to explore the influence of microbial composition on asthma pathogenesis, especially in the lung, to subsequently refine treatment regimens that can prevent airway diseases (Hufnagl et al., 2020).

### 6.2 Lung microbiome and cystic fibrosis

An important element in the pathophysiology of CF lung disease is airway inflammation. Studies anticipate that the microbiota would either impact or be affected by airway inflammation if it were to play a part in cystic fibrosis lung disease (Linnane et al., 2021). Studies confirm that the correlation is inconsistent across multiple inflammatory markers and different measures of diversity, as evaluated by NE and IL-8. Those markers are increasing with the degree of inflammation, but also in terms of biodiversity. There was no discernible impact of antibiotic exposure on the microbiota in the lower airways.

Although Zemanick et al. did demonstrate a strong link between antibiotic use and decreased diversity, a sizable fraction of the study’s population was taking antibiotics because the BALs were carried out for clinical purposes in symptomatic cases (Zemanick et al., 2017). Pittman et al. concluded that newborns receiving anti-Staphylococcal drugs had less diversity. Researchers used amoxicillin-clavulanate, a broad range antibiotic, which may interfere with many other species of bacteria within the lower respiratory tract (Pittman et al., 2017).

### 6.3 Lung microbiome and hematological malignancies

The lung microbiota and demographic features of children with lower respiratory tract infections (LRTIs) and hematological malignancies were compared to those of children with LRTIs and no malignancies. Studies observed significant differences in the red blood cell count, hemoglobin, platelet count, C-reactive protein, ratio of positive culture other than bronchoalveolar lavage fluid (BALF), and hospitalization course between the two groups that had identical age, weight, height, gender, and predisposing antibiotics course prior to hospitalization. All BALF samples from the two groups’ microbiomes revealed a markedly reduced diversity of α and β, a markedly enhanced function in infectious illness (bacteria/parasites), and treatment resistance in LRTI children with hematological malignancies (Pittman et al., 2017; Zemanick et al., 2017).

Authors found a greater percentage of *Proteobacteria* at the phylum level, a significantly lower proportion of *Firmicutes*, *Bacteroidota*, *Actinobacteriota*, and an evidently higher proportion of *Parabacteroides*, *Klebsiella*, *Grimontia*, *Escherichia, Shigella*, and unclassified *Enterobacteriaceae* at the genus level compared to the control group (LRTI children without any hematological malignancies). Additionally, it was discovered that hospitalization course was significantly correlated negatively with α diversity (Shannon), β diversity (Bray Curtis dissimilarity), *Proteobacteria* at the phylum level, unclassified *Enterobacteriaceae*, and *Escherichia, Shigella* at the genus level, while *Firmicutes* at the phylum level established a positive correlation with hospitalization course (Zhang et al., 2022).

Based on the results of various research, it is anticipated that the lung microbiome may emerge as a valuable biomarker for respiratory disorders in clinical settings. The lung microbiome may contain information that is both diagnostic and predictive. The ultimate goal of lung microbiome research is precision medicine, which involves identifying critical diagnostic or therapeutic traits that influence clinical outcomes.

## 7 Conclusions

The microbiome in bronchiectasis is significantly less explored than in other lung illnesses such as cystic fibrosis and chronic obstructive pulmonary disease; much of the evidence comes from the BLESS project. Whereas the significance of the microbiome in bronchiectasis remains unclear, existing data show lung bacterial communities dominated by *Haemophillus*, *Pseudomonas*, and *Streptococcus*, with significant interindividual variability and intraindividual stability. Frequent exacerbations and more severe disease have been shown to be associated with *Pseudomonas* and *Haemophillus* dominating microbiomes.

Additional research is needed to broaden our understanding of the microbiome in many patient groups, both stable and exacerbated and in response to various medications. These data should be used in clinical trials to identify populations of patients which have a greater probability to respond to specific medications or to guide more personalized treatment approaches.

## Author contributions

AA: Conceptualization, Investigation, Writing – original draft. AL: Conceptualization, Investigation, Writing – original draft. MA: Investigation, Software, Writing – original draft. IS: Investigation, Methodology, Writing – original draft. AM: Investigation, Software, Writing – original draft. VL: Project administration, Supervision, Writing – review & editing. EM: Validation, Visualization, Writing – review & editing. AN: Validation, Visualization, Writing – review & editing. RT: Validation, Visualization, Writing – review & editing. DM: Validation, Writing – review & editing. CM: Validation, Writing – review & editing, Software. DS: Writing – review & editing, Validation, Visualization. II: Investigation, Methodology, Writing – review & editing.
